# Mitigation of Gas Porosity in Additive Manufacturing Using Experimental Data Analysis and Mechanistic Modeling

**DOI:** 10.3390/ma17071569

**Published:** 2024-03-29

**Authors:** Satyaki Sinha, Tuhin Mukherjee

**Affiliations:** Department of Mechanical Engineering, Iowa State University, Ames, IA 50011, USA; satty51@iastate.edu

**Keywords:** laser powder bed fusion, 3D printing, convective flow, buoyancy, Stokes law, gas porosity index, stainless steel 316, Ti-6Al-4V, Inconel 718, AlSi10Mg

## Abstract

Shielding gas, metal vapors, and gases trapped inside powders during atomization can result in gas porosity, which is known to degrade the fatigue strength and tensile properties of components made by laser powder bed fusion additive manufacturing. Post-processing and trial-and-error adjustment of processing conditions to reduce porosity are time-consuming and expensive. Here, we combined mechanistic modeling and experimental data analysis and proposed an easy-to-use, verifiable, dimensionless gas porosity index to mitigate pore formation. The results from the mechanistic model were rigorously tested against independent experimental data. It was found that the index can accurately predict the occurrence of porosity for commonly used alloys, including stainless steel 316, Ti-6Al-4V, Inconel 718, and AlSi10Mg, with an accuracy of 92%. In addition, experimental data showed that the amount of pores increased at a higher value of the index. Among the four alloys, AlSi10Mg was found to be the most susceptible to gas porosity, for which the value of the gas porosity index can be 5 to 10 times higher than those for the other alloys. Based on the results, a gas porosity map was constructed that can be used in practice for selecting appropriate sets of process variables to mitigate gas porosity without the need for empirical testing.

## 1. Introduction

Laser powder bed fusion (LPBF) additive manufacturing is capable of printing 3D parts of a wide variety of steels and alloys of nickel, titanium, and aluminum for the aerospace, healthcare, automotive, and energy industries [1,2,3,4,5]. A laser beam selectively scans closely packed layers of powders and creates a molten pool, which after solidification, forms the part. Gas bubbles can originate inside the molten pool from shielding gas, metal vapors, and gases trapped inside powders during atomization [1]. If these gas bubbles are unable to escape from the molten pool before solidification, they can result in gas porosity inside the printed parts [6,7]. Gas porosities can significantly degrade the tensile [8,9,10,11,12,13] and fatigue properties [14,15,16,17] of parts. For example, the presence of porosity can increase the plastic strain during the tensile test to the point where additional plastic deformation is restricted to a smaller cross-sectional area or a region that was not work-hardened [11]. Because of this, tensile properties, such as ultimate tensile strength, are significantly reduced. Porosity is also a determining factor in the case of fatigue performance, as it can serve as a site for the initiation of fatigue cracks [14,15,16,17]. Therefore, mitigation of gas porosity is needed to improve the mechanical properties, reliability, and serviceability of metallic parts made by LPBF.

Several attempts have been made to mitigate gas porosity in LPBF parts (Table 1) using experimental techniques [18,19,20,21,22,23,24,25], mechanistic modeling [26,27,28,29,30,31,32,33,34], and machine learning [35,36,37,38,39,40,41]. However, experimental trial-and-error to adjust many process variables for reducing gas porosity is expensive and time-consuming. In addition, this trial-and-error approach does not always guarantee achieving an optimized set of process variables. Post-processing techniques [19,21,23], such as hot isostatic pressing, can reduce porosity but significantly add cost. Numerical models [26,27,28,29,30,31,32,33,34] have been developed to predict the formation of gas porosity by capturing the underlying physics. However, these models are computationally intensive and often difficult to use in real time. Machine learning models [35,36,37,38,39,40,41] can be used in real-time; however, they are often unable to capture the important physical factors causing porosity and need a large volume of high-quality data for a reliable prediction. Thus, the existing approaches (Table 1) using experimental, modeling, and machine learning methods are inadequate to reduce gas porosity in LPBF. Therefore, what is needed and currently unavailable is an integrated theoretical and experimental framework that can identify all important physical factors causing gas porosity and combine them in a comprehensible manner to predict and control the pore formation in LPBF of diverse metallic materials. This article aims to address that need.

Here, mechanistic modeling and experimental data analysis were combined to predict and control gas porosity during LPBF of stainless steel 316, Ti-6Al-4V, Inconel 718, and AlSi10Mg that are commonly used in the aerospace, automotive, healthcare, and energy industries. First, the important physical factors that impact the formation of gas porosity were calculated for a broad range of processing conditions. The computed results were rigorously tested against independent experiments. The results were aimed at providing a detailed, comprehendible scientific insight into the pore formation in LPBF. We used the modeling and experimental data to derive a verifiable, user-friendly, dimensionless gas porosity index to predict both the occurrence and amount of gas pores in LPBF parts. Finally, we constructed a process map to help engineers select appropriate processing conditions to mitigate gas porosity. The process map can also provide an in-depth scientific understanding of the effects of LPBF processing conditions on porosity formation. Although the results reported here are for the LPBF process, the methodology can be extended to mitigate gas porosity in laser and electron beam-directed energy deposition as well as in wire arc additive manufacturing processes.

## 2. Methodology

**Figure 1:**
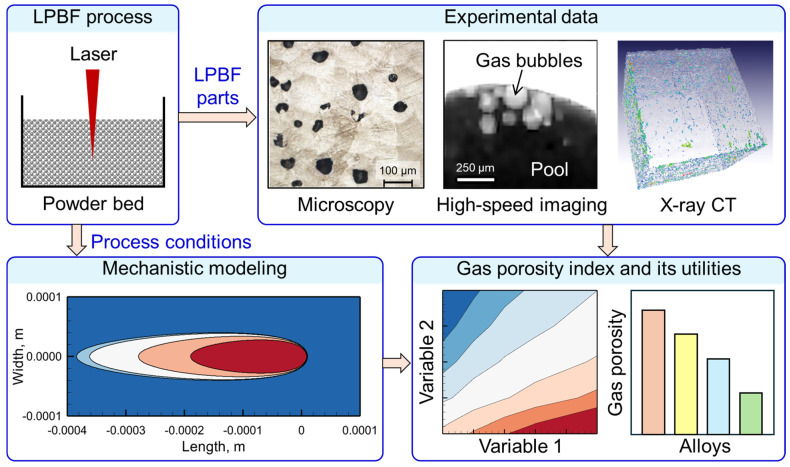
A schematic representation of the methodology used in this work. A combined approach of using mechanistic modeling and analysis of experimental data was implemented to derive and use a gas porosity index for predicting and controlling gas porosity during LPBF of common alloys. The pictures inside the “Experimental data” box are adapted from [7,61,62]. Figures taken from open-access articles [7,61] are under the terms and conditions of the Creative Commons Attribution (CC BY) license. The figure taken from [62] is under the permission obtained from Elsevier.

In this work, a combined approach (Figure 1) of using mechanistic modeling and analysis of experimental data on the occurrence of gas porosity in LPBF parts of various common alloys was used. First, a mechanistic model [42,43] of the LPBF process was tested, calibrated using experimental results, and used to compute temperature fields and molten pool geometry. Then, experimental data [44,45,46,47,48,49,50,51,52,53,54,55,56,57,58,59,60] on porosity during LPBF of stainless steel 316, Ti-6Al-4V, Inconel 718, and AlSi10Mg were collected from the literature. The results from the well-tested model were used to derive and calculate a gas porosity index corresponding to all experimental cases. The mechanistic model, calculation of the gas porosity index, and collection and analysis of experimental data are explained in detail below.

### 2.1. Assumptions

The following simplifying assumptions were made for the mechanistic model to make the calculations of temperature and molten pool geometry tractable:

(1) The laser beam moves at a constant speed in the same direction on a straight path relative to the substrate. The model assumed a quasi-steady state of heat conduction [1,43] where the coordinate along the scanning direction was transformed [1] to capture the effect of scanning speed.
(2) The laser beam energy was assumed to be focused at a point on the upper surface of the deposit and was applied at a uniform rate [1,43]. It was assumed that the width of the substrate is significantly larger than the track width, and the substrate is much thicker than the depth of the molten pool [1].
(3) The effects of the convective flow of molten metal, mainly driven by the spatial gradient of surface tension and buoyancy [1,63] on temperature fields were neglected. Heat losses from the surface through convection and radiation [1] were disregarded.
(4) Thermophysical properties of alloys were considered to be temperature-independent.
(5) The values of the laser absorptivity were assumed to be constant for a given alloy, even though it is expected to be somewhat influenced by other factors such as processing parameters, the presence of oxide and other surface impurities, surface roughness, and gas composition above the molten pool [64].
(6) Only conduction mode [2,3] LPBF was considered. Therefore, gas bubbles from unstable keyholes and resulting keyhole porosities [65,66] are not within the scope of this work.
(7) Effects of gas dissolution [1] in the liquid metal controlled by activity and partial pressure of gas were ignored by assuming the nucleation of gas bubbles on the solidifying interface. The bubble size was estimated by a pressure balance that ignored the effects of the coalescence of bubbles.

The results from the mechanistic model were compared with a multi-physics, 3D, transient heat transfer and fluid flow model of LPBF to prove that the assumptions are valid. The comparison results are reported in the Supplementary File.

### 2.2. Calculations of Temperature and Molten Pool Geometry

A mechanistic model of LPBF was used to compute the temperature distributions and the molten pool dimensions using process variables such as laser power, scanning speed, and preheat temperature, as well as alloy properties such as density, thermal conductivity, and specific heat as inputs. The thermophysical properties [2,3,67] of the alloys used in the model are reported in the Supplementary File. Under the same processing conditions, the molten pool shape and size for different alloys can significantly vary depending on these properties [42]. The temperature (T) at any location of the part can be expressed as [43,68]:

(1) $$T=T_{0}+\frac{Q}{2\pi \;k_{eff}\;\xi }\exp[-\frac{V(\xi +x)}{2\alpha_{eff}}]$$

where $T_{0}$ indicates the initial or preheat temperature, Q is the laser power absorbed, $k_{eff}$ is the effective thermal conductivity of the powder bed, V is the scanning speed, $\alpha_{eff}$ is the effective thermal diffusivity of the powder bed, ξ is the distance from the laser beam axis, and x represents the coordinate along the scanning direction. The effective powder bed thermophysical properties depend on the properties of both the metal powders and shielding gas [2]. The shielding gas trapped between the closely packed powders and the powder bed’s packing efficiency determine the effective thermo-physical properties of the packed powder bed [69]. The traditional solution of heat conduction equation was based on the properties of solid materials [70]. However, in this work, we modified the solution by considering the effective powder bed properties (Equation (1)). The effective thermal diffusivity ($\alpha_{eff}$) of the powder bed in Equation (1) is represented as [3]:

(2) $$\alpha_{eff}=\frac{k_{eff}}{{Cp}_{eff}\;\rho_{eff}}$$

where $k_{eff}$ is the effective powder bed thermal conductivity (Equation (1)). ${Cp}_{eff}$ and $\rho_{eff}$ are the effective specific heat and density of the powder bed, respectively. The effective properties are represented as [3]:

(3) $$k_{eff}=k_{s}\eta +k_{g}\left(1-\eta \right)$$

(4) $${Cp}_{eff}=\frac{\left((\rho_{s}\eta {Cp}_{s})+(\rho_{g}(1-\eta ){Cp}_{g})\right)}{\rho_{s}\eta +\rho_{g}(1-\eta )}$$

(5) $$\rho_{eff}=\rho_{s}\eta +\rho_{g}(1-\eta )$$

where η is the powder bed packing efficiency. In Equations (3)–(5), the suffix ‘s’ and ‘g’ represent the property values for the solid alloy and shielding gas, respectively. The thermophysical properties [2,3,67] of solid alloys and shielding gas [3] and packing efficiency are reported in the Supplementary File.

From the computed temperature field (Equation (1)), the dimensions of the molten pool were extracted by tracking the solidus isotherms of alloys. Calculated temperature and molten pool dimensions were used to derive and compute a gas porosity index for predicting and controlling gas porosity in LPBF, as discussed below.

### 2.3. Gas Porosity Index and Its Calculations

**Figure 2:**
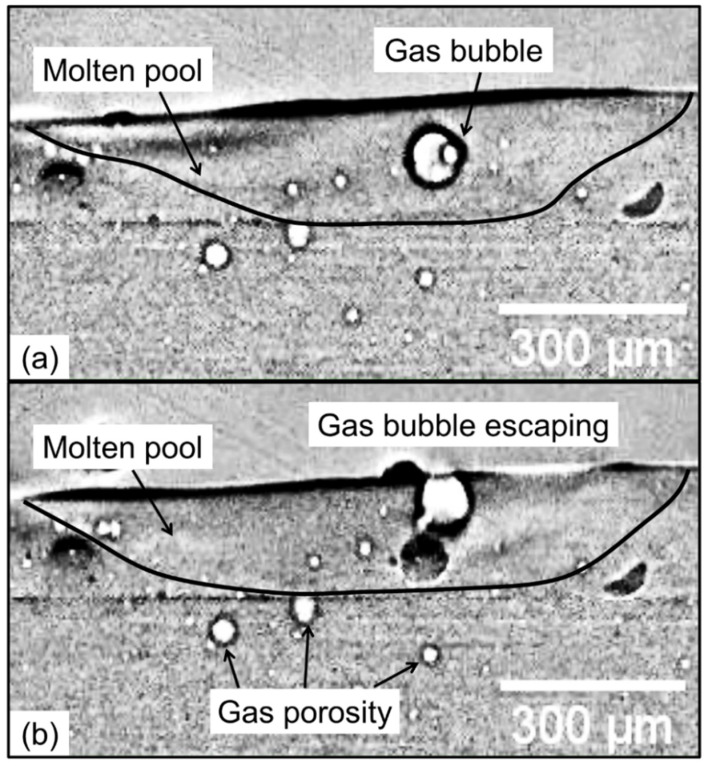
In situ high-speed synchrotron X-ray images showing the formation and dynamics of gas bubbles during directed energy deposition of a nickel-based superalloy. Side views of the molten pool are shown. (**a**) Gas bubbles inside the molten pool. (**b**) Gas bubbles are escaping from the molten pool. The figure is adapted from [7]. Figures are taken from an open-access article [7] under the terms and conditions of the Creative Commons Attribution (CC BY) license.

High-speed imaging [7] of both fusion welding and additive manufacturing processes has revealed that the gas bubbles formed inside the molten pool can result in porosity. For example, Figure 2 shows the presence of gas bubbles inside the molten pool. Gas porosities occur if the gas bubbles are unable to escape out of the molten pool before solidification. Therefore, the time needed for a gas bubble to rise and escape out of the molten pool and the solidification time of the pool are two key time factors affecting the formation of gas porosity. Here, the gas porosity index (τ) was defined as the ratio of the time to rise of the gas bubble ($T_{R}$) to the time to solidify ($T_{S}$) of the molten pool as:

(6) $$\tau =T_{R}/T_{S}$$

A high value of τ indicates that a gas bubble takes a long time to escape from the molten pool before solidification resulting in a high susceptibility to gas porosity. The index also captures the effects of process variables and alloy properties on porosity [71,72,73]. The two aforementioned times were calculated based on the results from the mechanistic model as explained below. A sample calculation of the gas porosity index is provided in the Supplementary File.

#### 2.3.1. Calculation of Time to Solidify

Time to solidify ($T_{S}$) indicates the time required for the solidification of the liquid metal pool and can be represented as:

(7) $$T_{S}=\frac{L}{V}$$

where L is the pool length estimated using the mechanistic model and V is the scanning speed. The time to solidify decreases at a higher scanning speed. This is because an increase in the scanning speed reduces the pool size, and the pool takes less time to solidify.

#### 2.3.2. Calculation of Time to Rise

Time to rise ($T_{R}$) indicates the time needed for a gas bubble to rise and escape out of the molten pool and can be written as:

(8) $$T_{R}=\frac{D}{u_{e}}$$

where the depth of the pool (D) was computed using the mechanistic model. $u_{e}$ is the escape velocity of the gas bubble. The calculation assumes that the nucleation of gas bubbles occurs on the solidifying interface near the bottom of the molten pool. It is well-known in the casting and fusion welding literature [74] that the velocity of a gas bubble inside a liquid depends on the size of the bubble. Therefore, we first calculated the size of the bubble by performing a pressure balance inside the liquid metal and used that to estimate the escape velocity as discussed below.

##### Calculation of Gas Bubble Size by Pressure Balance

The pressure inside a stable gas bubble ($P_{i}$) is equal to the sum of the surface tension pressure ($P_{s}$) and the liquid pressure ($P_{l}$) as [75]:

(9) $$P_{i}=P_{s}+P_{l}$$

Surface tension pressure ($P_{s}$) is given by [75]:

(10) $$P_{s}=\frac{2\sigma }{r}$$

where σ is the surface tension of the molten alloy and r is the radius of a spherical gas bubble. The liquid pressure ($P_{l}$) is represented as a summation of the atmospheric pressure ($P_{a}$) and the pressure due to the height of the liquid (ρgD) as:

(11) $$P_{l}=P_{a}+\rho gD$$

where ρ, g, and D are the density of the liquid metal, the acceleration due to gravity, and pool depth. For a tiny pool in LPBF, ρgD is negligible. Therefore,

(12) $$P_{l}=P_{a}$$

By using Equation (9) and the ideal gas law, the value of the radius of a spherical gas bubble (r) can be calculated as:

(13) $$\frac{4}{3}\pi r^{3}\left[P_{a}+\frac{2\sigma }{r}\right]=RT$$

where R is the universal gas constant and T is the solidus temperature of an alloy.

##### Escape Velocity of Gas Bubbles

Since the density of gas is much lower than that of the molten liquid, gas bubbles tend to rise inside the liquid pool due to the buoyancy. For small Reynolds numbers, the rising velocity of gas bubbles can be represented by the Stokes law [74]. Here the flow of the molten metal is assumed to be laminar with a low Reynolds number. Therefore, the approximate rising velocity of spherical bubbles called the Stokes velocity ($u_{s}$) is given by [76]:

(14) $$u_{s}=\frac{2}{9}\frac{r^{2}\Delta \rho g}{\mu }$$

where r is the radius of the gas bubble calculated using Equation (13), Δρ is the difference in density between the gas and the molten liquid, g is the acceleration due to gravity, and μ is the viscosity of the liquid. The possibility of bubbles escaping from the fusion zone is increased by the low liquid viscosity and large bubble radius [76].

It is well known [1] that the convective flow of liquid metal is mainly driven by the spatial gradient of surface tension (Marangoni effect) and buoyancy. High-speed imaging has shown that the Marangoni effect on the gas bubble dynamics is important only near the pool surface [77]. However, a gas bubble often nucleates near the bottom surface, where buoyancy may play a more crucial role [78]. Because of the greater dominance of the buoyancy force, we compute the convective velocity [79] as:

(15) $$u_{c}=\sqrt{g\beta \Delta TD}$$

where g is the acceleration due to gravity, β is the coefficient of volumetric expansion, ΔT is the temperature gradient, and D is the depth of the pool computed using the mechanistic model. This convective velocity of liquid metal accelerates the escape velocity of the gas bubbles. Therefore, the escape velocity of gas bubbles ($u_{e}$) is the summation of two aforementioned velocities [76] as:

(16) $$u_{e}=u_{S}+u_{c}$$

The value of the escape velocity is used in Equation (8) to estimate the time to rise of the gas bubble.

### 2.4. Data Collection and Analysis

A total of 93 sets of data on gas porosity formation for four alloys at various processing conditions were collected from the literature [44,45,46,47,48,49,50,51,52,53,54,55,56,57,58,59,60]. Among the 93 sets of data, 60 cases had gas pores and 33 cases were without experimentally detected gas pores. The mechanistic modeling was performed for all 93 cases to calculate the gas porosity index. Using this data, the gas porosity index was tested for the four alloys and the range of variables provided in Table 2. The Supplementary File contains the values of all variables corresponding to the 93 experimental cases for which calculations were performed.

## 3. Results and Discussion

### 3.1. Comparison of Molten Pool Geometry of Four Alloys

**Figure 3:**
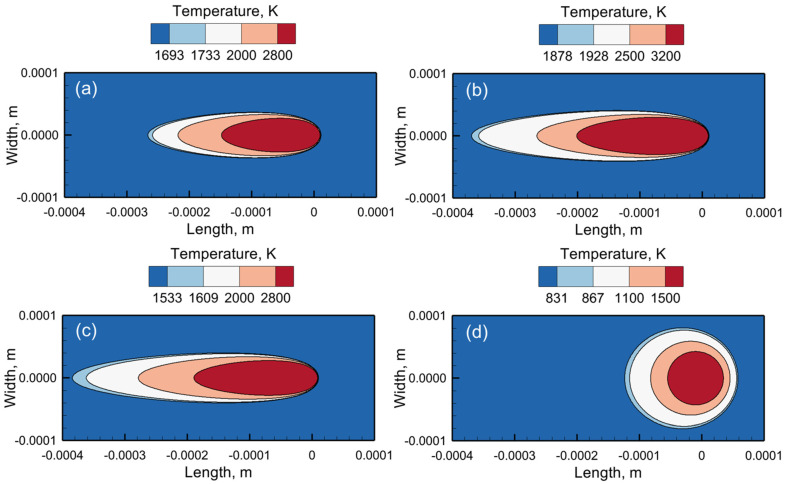
Computed temperature fields and molten pool geometries on the deposit top surface during LPBF of (**a**) Stainless steel 316, (**b**) Ti-6Al-4V, (**c**) Inconel 718, and (**d**) AlSi10Mg using 300 W laser power and 1250 mm/s scanning speed. The results correspond to the location of the laser beam axis at (0,0). The scanning direction is from left to right (along the positive length axis) in all figures. In each figure, the values of the isotherms can be read from the corresponding contour legends.

Both the time needed for a gas bubble to rise and escape out of the molten pool and the solidification time of the pool that determines the gas porosity (Section 2.3) are significantly affected by molten pool dimensions. Since different alloys exhibit a wide variety of molten pool geometries [64], it is important to compare them under the same processing conditions. Figure 3a–d shows the computed temperature distribution on the deposit top surface for the four common alloys. The region surrounded by the isotherm of solidus temperature represents the molten pool. The fusion zone is enclosed by the liquidus isotherm of an alloy. The sky-blue area between the liquidus and solidus isotherms in each figure represents the mushy zone. The laser beam scans from the left to the right direction. Therefore, the molten pool is elongated in the opposite direction of the scanning direction.

The different shapes of the molten pool are due to the variation in the thermophysical properties of the four alloys. The molten pools for SS 316, Ti-6Al-4V, and Inconel 718 exhibit a tear-dropped and elongated shape due to rapid scanning. The molten pool for AlSi10Mg is elliptical (Figure 3d) because the heat distribution is nearly uniform in all directions, attributed to its high thermal diffusivity. Consequently, it has a large width and a short length. This elliptical pool has the largest volume compared to the molten pools of the other three alloys. In addition, the temperature inside the AlSi10Mg molten pool is the lowest among the four alloys due to its very high thermal diffusivity. Ti-6Al-4V shows a larger liquid pool (Figure 3b) than SS 316 (Figure 3a) because of its lower density. In addition, a larger difference between the liquidus temperature and solidus temperature of Inconel 718 results in a very elongated mushy zone and molten pool (Figure 3c). Although Ti-6Al-4V and Inconel 718 exhibit similar pool sizes, the temperature inside the molten pool of Ti-6Al-4V is higher due to its lower density than Inconel 718.

**Figure 4:**
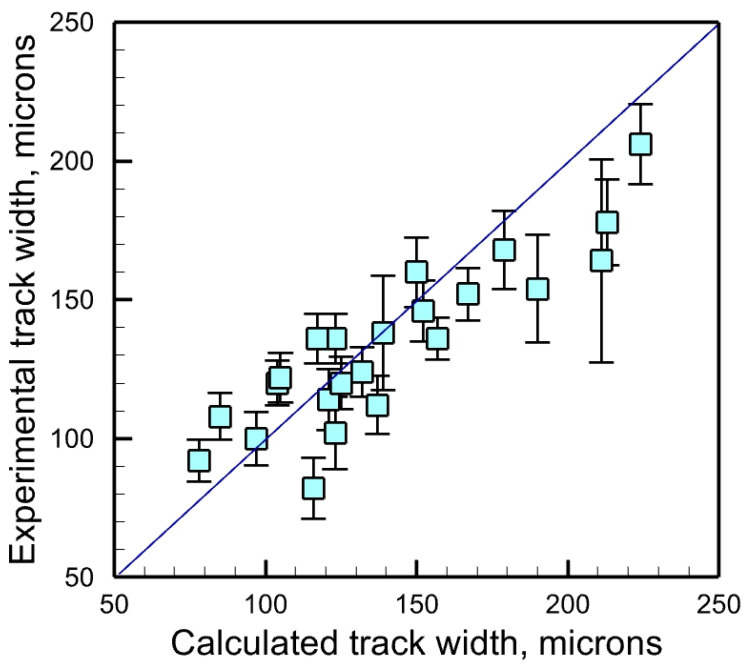
Comparison between the calculated and the experimentally measured [45] track width of stainless steel 316 deposits made by LPBF at different laser powers and scanning speeds. The RMSE value for the experimental width and calculated width is 35.2 microns. The average of the error bars present is calculated as 27.7 microns.

Figure 4 compares the calculated and experimentally measured [45] track width of stainless steel 316 deposits made by LPBF at different laser powers and scanning speeds. The proximity of the data points to the 45° line indicates that the computed results agree well with the experiments. The track width for each condition was measured five times along the track, and an average value was reported. The error bars represent the standard deviations. The RMSE value of the prediction is 35.2 microns, which is very similar to the average of the error bars present in the experimental data (27.7 microns). It indicates that the error in prediction is heavily influenced by the uncertainties in the measurement. In addition, several simplifying assumptions in the calculations (Section 2.1) have also contributed to the error. The reasonably good match between the computed and experimental results gives us the confidence to use the mechanistic model to compute the gas porosity index for different alloys and processing conditions.

### 3.2. Prediction of Porosity Using Gas Porosity Index

A gas porosity index (τ) is an easy-to-use, verifiable, and dimensionless indicator (see Section 2.3) that can predict gas porosity defects. There are two main utilities of the gas porosity index. First, it can predict if porosity will form or not under a given set of processing conditions for a particular alloy. Second, if porosity forms, the gas porosity index can provide an approximate quantitative idea of its amount. These two utilities are discussed below.

**Figure 5:**
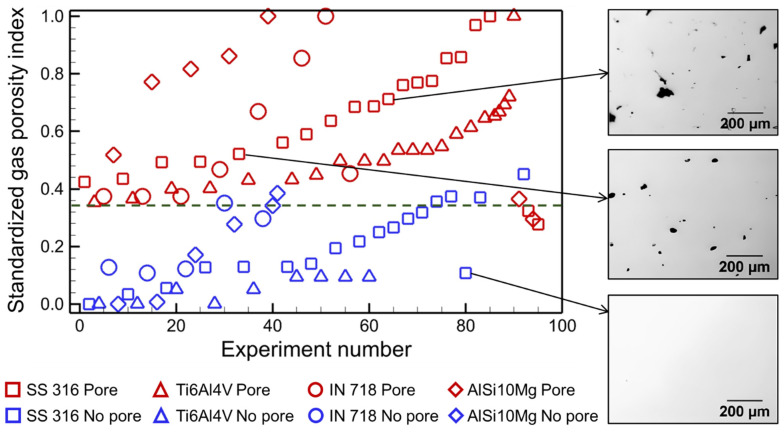
Gas porosity index to predict porosity in LPBF. The figure displays the index values for the 93 experimental cases. The index values are standardized to plot the data for all alloys under the same scale. The threshold value which delineates the pore and no pore cases is shown by a horizontal dashed line. Three optical micrographs [45] with the presence and absence of pores for LPBF of stainless steel 316 are shown corresponding to three experimental data points. The micrographs are taken from an open-access article [45] under the terms and conditions of the Creative Commons Attribution (CC BY) license.

Figure 5 analyzes the values of the gas porosity index for the 93 experimental cases (see Section 2.4) for four alloys. The values of the gas porosity index are provided in the Supplementary File. We noticed that the gas porosity index values of the four alloys vary widely, primarily due to the differences in their thermophysical properties and pool dimensions (Figure 3). For an easy comparison, we put all values of the gas porosity index on a consistent scale by standardizing the values. For standardization, the difference between each value and the minimum value is divided by the range of the index value for each alloy. The figure shows that the gas porosity index can accurately delineate the cases with pores from the cases where no pores were observed experimentally with an accuracy of 92%. The threshold value delineating the two cases is an essential point of reference for figuring out if the manufactured parts have porosity or not. Three optical micrographs that correlate to particular data points in the figure demonstrate the usefulness of the gas porosity index in accurately predicting the occurrence of gas porosity. The results show that the proposed methodology is consistent with the independent experiments conducted at various processing conditions using different LPBF machines and materials. Using the index, it is possible to identify the appropriate combination of the process variables to minimize porosity.

**Figure 6:**
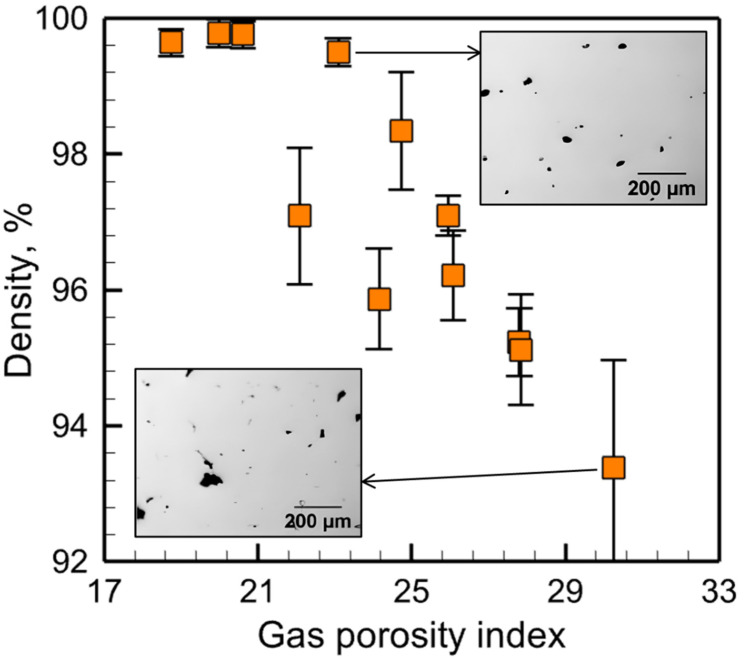
Variation in the volume percentage of density in stainless steel 316 parts made by LPBF with the computed gas porosity index. The reported density values [45] are the average of five measurements and the error bars represent the standard deviation of it. Two optical micrographs [45] with different amounts of pores for LPBF of stainless steel 316 are shown corresponding to two data points of gas porosity index. The micrographs are taken from an open-access article [45] under the terms and conditions of the Creative Commons Attribution (CC BY) license.

If porosity is expected to form, the gas porosity index can provide an approximate quantitative idea of its amount. Figure 6 shows that the part density decreases as the amount of porosity increases at a higher value of the gas porosity index. In the figure, the percentage of density is plotted instead of porosity because the part density is a more intuitive parameter and easy to measure during experiments. The top right optical micrograph corresponds to a part having a density of 99.5%, which indicates that the part has low porosity, consistent with its gas porosity index value of about 23.09. In contrast, the other optical micrograph shows a density of about 93.39% and a higher gas porosity index of 30.26.

This result has important implications for manufacturing processes that depend on attaining accurate part densities. In order to reduce the likelihood of porosity in the finished product, the result emphasizes the significance of predicting the gas porosity index. Manufacturers can optimize their process conditions to obtain desired part densities and improve overall product quality and performance without any time-consuming and expensive trial-and-error.

### 3.3. Relative Susceptibility of Alloys to Gas Porosity

**Figure 7:**
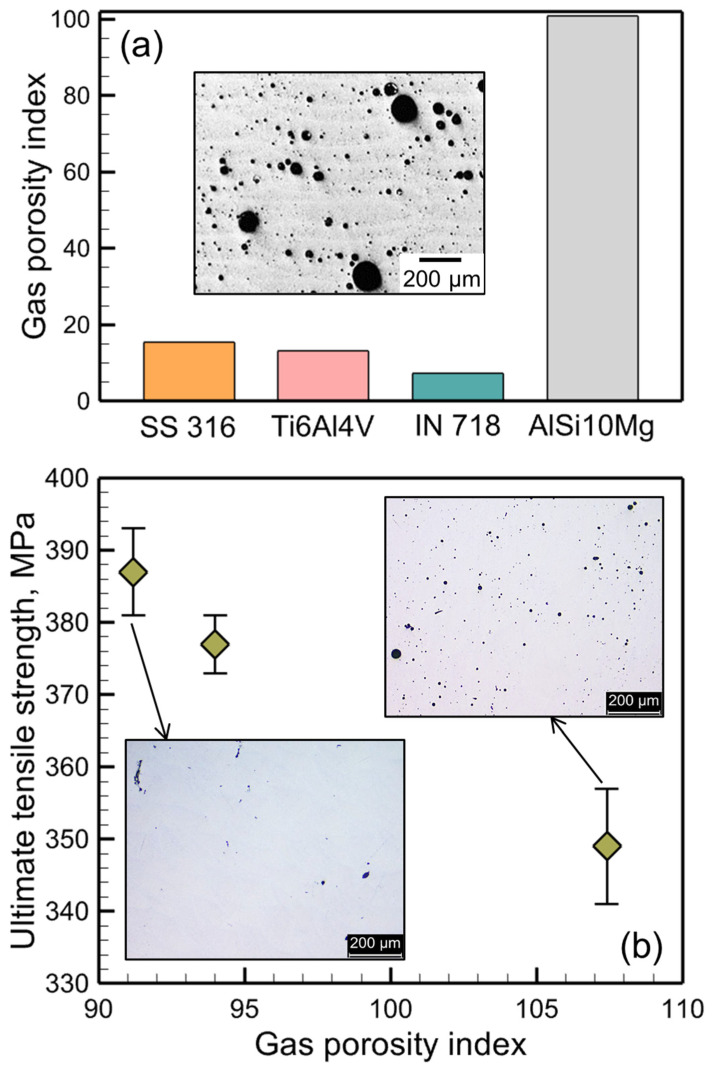
(**a**) Relative susceptibilities of four commonly used alloys to gas porosity evaluated by the computed values of the gas porosity index during LPBF using 300W laser power and 1250 mm/s scanning speed. The inset shows an optical micrograph [82] of an AlSi10Mg part made by LPBF containing a significant amount of gas pores. The micrograph is taken from [82] with permission from Elsevier. (**b**) A reduction in the ultimate tensile strength of AlSi10Mg parts made by LPBF due to the presence of gas porosity. The insets show the optical micrographs [83] of the samples containing different amounts of porosity. The plot is made based on the experimental data reported in [83] where heat input was varied to produce parts with different amounts of porosity. Corresponding values of the gas porosity index were calculated. The micrographs are taken from a thesis [83] available in the public domain.

The gas porosity index provides a quantitative scale for estimating and comparing the relative vulnerabilities of different alloys to gas porosity. Figure 7a compares four commonly used alloys based on their relative susceptibility to gas porosity under a given set of processing conditions. A long, elongated, tear-drop-shaped molten pool for Inconel 718 (Figure 3) allows the gas bubbles a long time to escape. Thus, Inconel 718 is less vulnerable to gas porosity. In contrast, gas bubbles need a long time to escape from a deep, hemispherical pool of AlSi10Mg. Thus, AlSi10Mg is the most susceptible to gas porosity among the four alloys. The high vulnerability of AlSi10Mg to gas porosity has been experimentally observed by many researchers [80,81]. For example, the inset in Figure 7a shows an optical micrograph [82] of an AlSi10Mg part made by LPBF. The part contains a very high amount of porosity that may lead to part rejection. For a different set of experiments [83] on the LPBF of AlSi10Mg, the gas porosity index values were calculated and reported in Figure 7b. A reduction in the ultimate tensile strength was found for the parts with high porosity, as indicated by a large value of the gas porosity index. The insets show the optical micrographs [83] of the samples containing different amounts of porosity. This result shows that the gas porosity index can also be beneficial to help engineers improve tensile properties by minimizing gas porosity. In addition, the index can be used to construct process maps for shop floor usage to reduce porosity in LPBF parts, as discussed below.

### 3.4. Gas Porosity Map

**Figure 8:**
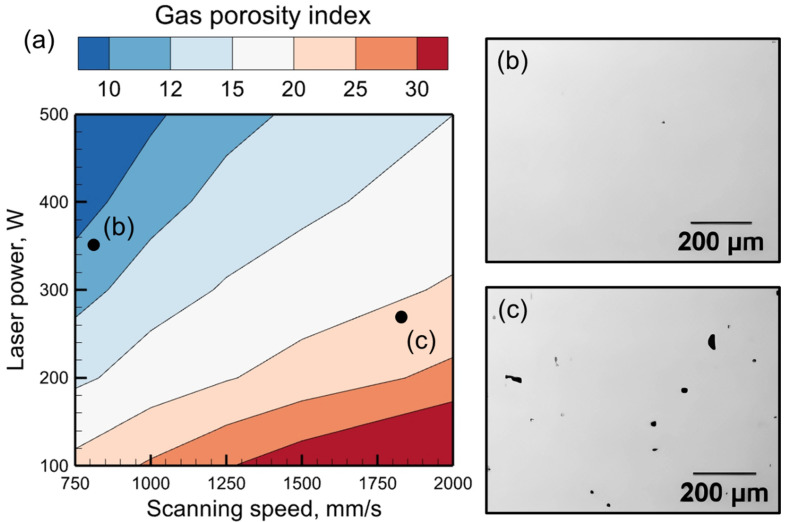
(**a**) A gas porosity map showing the variations in gas porosity index with laser power and scanning speed during LPBF of stainless steel 316. (**b**,**c**) Two micrographs [45] indicate the presence and absence of porosity for the corresponding conditions in (**a**). The micrographs are taken from an open-access article [45] under the terms and conditions of the Creative Commons Attribution (CC BY) license.

The calculated values of the gas porosity index for different processing conditions and alloys can be utilized to construct gas porosity maps. These maps can indicate the optimum process windows for mitigating gas porosity. Figure 8a shows a gas porosity map, where the contour values represent the magnitude of the gas porosity index during LPBF of stainless steel 316. It is evident that high laser power and slow scanning are beneficial for reducing porosity. In contrast, rapid scanning can lead to fast solidification, resulting in the entrapment of gas bubbles and porosity.

Two micrographs (Figure 8b,c) have been provided to test the map against experimental results [45]. If a laser power of 350 W and a scanning speed of 800 mm/s (Figure 8b) are used, then a dense part is obtained with almost no porosity with a gas porosity index of 17.05. In contrast, a laser power of 275 W and a scanning speed of 1800 mm/s result in a part (Figure 8c) that contains porosity and a gas porosity index of 20.61. This validates the gas porosity map for stainless steel 316 within the range of the process parameters considered in this work. Such maps, when rigorously verified against independent experimental results for various alloys and a wide range of processing conditions, can be made available for real-time prediction of pore formation on the shop floor.

## 4. Summary and Conclusions

In this work, a combination of mechanistic modeling and experimental data analysis was implemented to derive an easy-to-use, verifiable, dimensionless gas porosity index. The index captured the effects of both process parameters and important alloy properties and included several dominant physical factors causing gas porosity. The results were tested for commonly used alloys: stainless steel 316, Ti-6Al-4V, Inconel 718, and AlSi10Mg. Below are the important findings:

(1) The integrated theoretical and experimental framework identified all important physical factors causing gas porosity. The dimensionless gas porosity index, which is the ratio of the time taken by a gas bubble to escape the molten pool to the time required to solidify the molten pool, delineated the experimental cases with pores from the cases where porosities were not observed with an accuracy of 92%. Higher values of the index indicate that the gas bubbles need a longer time to escape from the molten pool, which increases their susceptibility to gas porosity. Experimental data proved that the number of pores increased at a higher value of the gas porosity index.

(2) Gas bubbles need more time to escape from a larger molten pool. Among the four alloys studied in this work, AlSi10Mg has the largest molten pool under the same processing conditions because of its lowest density. Thus, AlSi10Mg is the most vulnerable to gas porosity among the four alloys. The values of the gas porosity index for AlSi10Mg are 5 to 10 times higher than those for the other alloys. The high susceptibility of AlSi10Mg to gas porosity has also been experimentally observed and reported in the literature. In contrast, an elongated molten pool allows more time for the gas bubbles to escape before they solidify. Inconel 718 exhibits the longest molten pool among the four alloys because it has a large difference between the liquidus and solidus temperatures. Thus, among the four alloys, Inconel 718 is the least susceptible to gas porosity.

(3) The gas porosity process map constructed here showed that for a particular alloy, less heat input at low laser power and fast scanning resulted in a small pool that solidified rapidly, prevented the gas bubbles from escaping, and made the part prone to gas porosity. These process maps, when rigorously tested against experiments, can be made available for shop-floor usage by selecting appropriate processing conditions to reduce porosity without the need for experimental trials.

## Figures and Tables

**Table 1:** Several existing approaches to reduce gas porosity in LPBF [18,19,20,21,22,23,24,25,26,27,28,29,30,31,32,33,34,35,36,37,38,39,40,41].

Approach	Alloy	Example	Ref.
Experimental approach	SS 316	Porosities were eliminated by changing energy density guided by X-ray CT, Optical Microscopy, and Archimedes method.	[16]
	SS 316	Porosities were successfully removed by wire electrical discharge polishing-based post-processing.	[17]
	SS 316	Adjustment of the process variables such as point distance, exposure time, and layer thickness during experiments lowered porosity.	[18]
	Ti6Al4V	Laser post-processing decreased gas pores which was confirmed by micro-CT examination.	[19]
	Ti6Al4V	Gas pores were eliminated by post-process hot isostatic pressing at different temperatures and pressures.	[20]
	AlSi10Mg	A low temperature (350 °C) hot isostatic pressing minimized gas porosity in the manufactured parts.	[21]
	Inconel 718	To reduce the gas porosity, different heat-treatment procedures including aging treatments were used.	[22]
	Inconel 718	Gas porosities were prevented by implementing a high-energy-intensity laser beam that resulted in a larger molten pool.	[23]
Mechanistic modeling approach	SS 316	Gas pores were eliminated by identifying appropriate conditions through thermodynamic calculations and genetic algorithms.	[24]
	SS 316	To reduce gas porosities laser power was varied guided by mechanistic modeling.	[25]
	SS 316	Porosities were successfully removed by varying the volumetric energy density assisted by a numerical model.	[26]
	SS 316	A calculation scheme was introduced that included normalized enthalpy and powder absorptivity measurements to decrease porosity.	[27]
	Ti6Al4V	Process maps were introduced that utilized normalized energy density to reduce porosity.	[28]
	Ti6Al4V	A dimensional analysis helped to reduce porosity and explained variability in defect behavior.	[29]
	AlSi10Mg	A molecular dynamics analysis showed that decreasing the hydrogen content and maximizing the cooling/heating times reduced porosity.	[30]
	AlSi10Mg	Thermal history and graph theory helped to identify appropriate conditions to reduce porosity.	[31]
	AlSi10Mg	Dynamics and mechanisms of pore motion were simulated using a multi-physics model to identify conditions for pore reduction.	[6]
	Inconel 718	A modeling technique was used that coupled laser powder interaction to examine spatter interaction and decreased porosity.	[32]
Machine learning approach	SS 316	A novel approach using thermography and deep learning was used to anticipate and reduce local porosity.	[33]
	SS 316	Improved Regression along with Convolutional Neural Networks were used to reduce porosity.	[34]
	Ti6Al4V	A deep learning architecture was employed using heat signals to predict and minimize porosity.	[35]
	Ti6Al4V	A deep learning technique was used for porosity reduction and monitoring that used Convolutional Neural Networks.	[36]
	AlSi10Mg	Effective reduction efforts were aided by porosity-type classification through machine learning.	[37]
	AlSi10Mg	Porosity was reduced through the use of Convolutional Neural Networks that were trained using the molten pool data.	[38]
	Inconel 718	Defect detection in SEM pictures was automated by deep learning, which promoted stochastic development and lowered porosity.	[39]

**Table 2:** Range of process parameters and thermophysical properties of alloys.

Parameters	Range
Laser power (W)	30–331
Scanning speed (mm/s)	50–3400
Pool length (micron)	149–2298
Pool width (micron)	64–664
Time to rise (ms)	5.15–69.75
Time to solidify (ms)	0.18–29.34
Thermal conductivity (W/m-K)	28.1–113.0
Specific heat (J/Kg-K)	409.6–2894.2
Viscosity (Kg/m-s)	0.0013–0.007
Surface tension (N/m)	0.82–1.82

## Data Availability

Data used in this research are available in the article and Supplementary File.

## Supplementary Materials

The following supporting information can be downloaded at: https://www.mdpi.com/article/10.3390/ma17071569/s1. Refs. [2,3,44,45,46,47,48,49,50,51,52,53,54,55,56,57,58,59,67,84] are cited in the Supplementary Materials.
