# Supplementary material for: Mitigation of Gas Porosity in Additive Manufacturing Using Experimental Data Analysis and Mechanistic Modeling
Source: Materials (Basel). 2024 Mar 29;17(7):1569. doi: 10.3390/ma17071569 (PMC11013015; doi:10.3390/ma17071569)
Supplement: Supplementary file 1 [file materials-17-01569-s001.zip › materials-2925063-supplementary.pdf]

Mitigation of Gas Porosity in Additive Manufacturing Using Experimental Data Analysis and Mechanistic Modeling

Satyaki Sinha and Tuhin Mukherjee *

Department of Mechanical Engineering, Iowa State University, Ames, IA 50011, USA; satty51@iastate.edu
*Correspondence: tuhinm@iastate.edu

Supplementary File includes:

Table S1, Thermophysical properties of the four alloys.

An example of calculation procedures: Time to Rise (T_R), Time to Solidify (T_S), and Gas Porosity Index (τ).

Table S2, Unprocessed process variables, gas property for 93 independent experimental data with defects and defect-free (gas pores and no gas pores) for four alloys.

Comparison with a multi-physics model: The temperature field and molten pool geometry calculated using the mechanistic model used in this work are compared with those obtained from a multi-physics numerical model.

Supplementary References, for more information about the gas porosity defects in additive manufacturing, peer-reviewed literature from which experimental data are taken, and mechanistic model calculations.

Table S1: Thermophysical properties [1-3] of the alloys used. Viscosity and surface tension are given at the liquidus temperature of alloys.

Properties	SS 316	Ti-6Al-4V	Inconel 718	AlSi10Mg
Solidus Temperature (K)	1693	1878	1533	831
Liquidus Temperature (K)	1733	1928	1609	867
Boiling Point (K)	3100	3133	3013	1380
Thermal Conductivity (W/m-K)	29.76	28.09	28.56	113.00
Specific Heat (J/Kg-K)	2894.2	524.6	409.6	565.3
Density (Kg/m^3)	7800	4000	8100	2670
Viscosity (Kg/m-s)	7×10^{-3}	4×10^{-3}	5×10^{-3}	1.3×10^{-3}
Surface Tension (N/m)	1.5	1.52	1.82	0.82
Powder Packing Efficiency	0.6	0.65	0.7	0.58

An example of calculation procedures:

Here we provide an example of calculation procedures of Time to Rise (T_R), Time to Solidify (T_S), and Gas Porosity Index (τ). These calculations are for the 93 experimental cases [4-20]. Since the calculation procedures are the same for all four alloys, we have taken into consideration the calculation for the stainless steel 316 as an example. For the data collected in this work, there are 60 gas pore defects and 33 no gas pore defects among the 93 data points from peer-reviewed papers.

Gas Pore Defects	No Gas Pore Defects	Total
60	33	93

Because the Time to Rise (T_R) and Time to Solidify (T_S) are calculated from the pool dimensions, the initial step is to calculate the molten pool dimensions for a particular power (P) and scanning speed (V). The calculation for the molten pool dimensions is done by using a mechanistic model.

We are using stainless steel 316 as an example calculation. We are taking the data reported in Figure 7 (a) of the main article for the example calculation. The dataset for it is given below:

Power (P) = 300 Watt
Scanning Speed (V) = 1250 m/s
Length (L) = 1349 μm = 0.001349 m
Width (W) = 150 μm = 0.00015 m
Depth (D) = (W/2) = 75 μm = 0.000075 m

Now time to solidify (T_S) can be calculated using Eq. 7 of the main article as,

$$T_S = \frac{0.001349}{1250}\ sec = \mathbf{0.0010792}\ \boldsymbol{sec}$$

Now we calculate the radius of the gas bubble (r) by balancing the pressure inside the molten pool using Eq. 13 of the main article.

We know, for 0.0224 m^3 = 1 mole

Therefore, $\frac{4}{3}\pi r^3$ m^3 = $(\frac{4}{3}\pi r^3 * \frac{1}{0.0224})$ mole = $0.029867\pi r^3$ mole

For stainless steel 316,

Surface Tension, σ = 1.5 N/m

Solidus temperature, T = 1693 K

Atmospheric Pressure, P_a = 1.013 * 10^5 Pascal = 101325 N/m^2

By substituting these values in Eq. 13 of the main article,

$$\frac{4}{3}\pi r^3 \left[101325 + \frac{2*1.5}{r}\right] = 0.029867\pi r^3 * R * T$$

$$\left[101325 + \frac{2*1.5}{r}\right] = 0.1862448 * 1693$$

Solving it we get **r = 0.0000297 m**

Next, we use the value of r in the Stokes Velocity equation (Eq. 14 of the main article) to calculate the velocity of the gas bubble. Difference in density between the molten SS 316 and the gas,

Δρ = (7800 – 0.974) Kg/m^3 = 7799.026 Kg/m^3 where $\rho_g = 0.974$ Kg/m^3

Viscosity of the alloy, μ = 0.007 Kg/m-s, acceleration due to gravity, g = 10 m/s^2

Now substituting the values in Eq. 14 of the main article,

$$u_s = \frac{2*(0.0000297)^2*7799.026*10}{9*0.007} = 0.0022283\ m/s$$

To find the escape velocity, we also need the convective velocity (u_c) using Eq. 15 of the main article. To do so, we also need the following alloy properties:

Co-efficient of volume expansion, β = $10^{-5}\ K^{-1}$

Depth of the molten pool, D = 0.000075 m

Acceleration due to gravity, g = 10 m/s^2

Temperature Gradient, $\Delta T = (\frac{T_{sol}+T_{boil}}{2} - T_{sol})$ = $(\frac{1693+3100}{2} - 1693)$ = 703.5 K

From Eq. 15 of the main article, we get:

$$u_c = \sqrt{(10 * 10^{-5} * 703.5 * 0.000075)} = 0.002297009\ m/s$$

Now, substituting the Stokes velocity and convective velocity in the equation of escape velocity, (Eq. 16 of the main article) we get,

$$u_e = u_s + u_c = (0.0022283 + 0.002297009)\ \text{m/s} = 0.004525309\ \text{m/s}$$

Therefore, the time to rise (T_R) is calculated as:

$$T_R = \frac{0.000075}{0.004525309}\ sec = \mathbf{0.016573454}\ \boldsymbol{sec}$$

Therefore, the gas porosity index (τ) = $\frac{Time\ to\ Rise\ (T_R)}{Time\ to\ Solidify\ (T_S)} = \frac{0.016573454}{0.0010792}$

Gas Porosity Index, τ = 15.35

Table S2: Process parameters and computed variables for 93 experimental cases [4-19].

No.	Alloy	Laser power (W)	Scan Speed (mm/s)	Length (micron)	Width (micron)	Depth (micron)	Time to Rise (s)	Time to Solidify (s)	Gas Porosity Index	Ref.
1	Stainless Steel 316	175	750	796	146	73	1.624E-02	1.060E-03	15.32	4
2		100	500	466	134	67	1.523E-02	9.320E-04	16.34	
3		100	750	460	110	55	1.310E-02	6.100E-04	21.48	
4		100	1000	458	96	48	1.180E-02	4.580E-04	25.76	
5		175	1000	793	128	64	1.470E-02	7.930E-04	18.54	
6		70	500	331	112	56	1.330E-02	6.640E-04	20.03	
7		50	500	240	94	47	1.161E-02	4.800E-04	24.19	
8		30	500	149	70	35	9.220E-03	2.980E-04	30.94	
9		200	1400	1008	114	57	1.350E-02	7.200E-04	18.75	5
10		200	1800	898	102	51	1.237E-02	5.000E-04	24.74	
11		200	2200	897	92	46	1.140E-02	4.100E-04	27.80	
12		200	2600	896	82	41	1.044E-02	3.450E-04	30.26	
13		275	1800	1232	120	60	1.401E-02	6.800E-04	20.60	
14		275	2200	1233	108	54	1.293E-02	5.600E-04	23.09	
15		275	2600	1230	100	50	1.220E-02	4.700E-04	25.96	
16		275	3000	1231	92	46	1.142E-02	4.100E-04	27.85	
17		350	2200	1563	122	61	1.419E-02	7.100E-04	19.99	
18		350	2600	1565	112	56	1.330E-02	6.020E-04	22.09	
19		350	3000	1565	104	52	1.256E-02	5.200E-04	24.15	
20		350	3400	1564	98	49	1.200E-02	4.600E-04	26.08	
21		125	400	582	168	84	1.800E-02	1.460E-03	9.36	
22		125	600	575	138	69	1.560E-02	9.600E-04	10.08	
23		125	800	571	120	60	1.401E-02	7.140E-04	10.55	
24		200	400	920	214	107	2.150E-02	2.300E-03	12.10	
25		200	600	912	174	87	1.850E-02	1.520E-03	12.17	
26		200	800	907	152	76	1.674E-02	1.134E-03	12.17	
27		200	1000	903	136	68	1.540E-02	9.030E-04	12.39	
28		200	1200	902	124	62	1.440E-02	7.500E-04	13.54	
29		275	600	1246	206	103	2.090E-02	2.080E-03	14.05	
30		275	800	1244	178	89	1.881E-02	1.560E-03	14.76	
31		275	1000	1238	160	80	1.740E-02	1.240E-03	15.09	
32		275	1200	1237	146	73	1.620E-02	1.030E-03	15.76	
33		275	1400	1237	136	68	1.540E-02	8.840E-04	16.25	
34		350	800	1576	204	102	2.080E-02	1.970E-03	17.05	
35		350	1000	1572	182	91	1.910E-02	1.572E-03	17.43	
36		350	1200	1570	164	82	1.771E-02	1.310E-03	17.71	
37		350	1400	1568	154	77	1.690E-02	1.120E-03	19.11	
38		350	1800	1565	136	68	1.540E-02	8.700E-04	19.63	
39	AlSi10Mg	360	1000	873	524	262	6.758E-02	8.700E-04	77.68	6
40		170	800	462	356	178	5.227E-02	5.760E-04	90.75	7
41		180	900	479	356	178	5.227E-02	5.320E-04	98.25	
42		190	1000	495	354	177	5.207E-02	4.950E-04	105.19	
43		200	1200	507	340	170	5.066E-02	4.225E-04	119.91	8
44		180	1400	454	302	151	4.670E-02	3.240E-04	144.14	7

45		190	1500	472	302	151	4.673E-02	3.150E-04	148.35	
46		200	1600	492	304	152	4.694E-02	3.075E-04	152.65	
47		381	1567	887	444	222	6.060E-02	5.700E-04	70.29	9
48		295	1167	720	434	217	5.968E-02	6.170E-04	70.91	
49		372	915	908	550	275	6.975E-02	9.920E-04	86.67	
50		321	1077	784	472	236	6.310E-02	7.300E-04	96.74	
51		299	1267	723	424	212	5.877E-02	5.700E-04	102.98	
52		325	851	811	524	262	6.758E-02	9.530E-04	107.04	
53	Inconel 718	95	200	650	222	111	1.470E-02	3.250E-03	4.52	10
54		95	800	623	112	56	8.350E-03	7.790E-04	10.72	
55		75	800	495	100	50	7.580E-03	6.188E-04	12.25	
56		75	1200	492	81	40.5	6.390E-03	4.100E-04	15.59	
57		75	1600	490	72	36	5.710E-03	3.060E-04	18.66	
58		75	2000	489	64	32	5.150E-03	2.445E-04	21.06	
59		120	800	784	126	63	9.220E-03	9.800E-04	9.41	
60		120	1200	782	104	52	7.840E-03	6.520E-04	12.02	
61		285	1000	1840	176	88	1.218E-02	1.840E-03	6.62	11
62		230	760	1492	180	90	1.241E-02	1.963E-03	6.32	
63		200	700	1302	176	88	1.218E-02	1.860E-03	6.55	
64		95	750	625	116	58	8.601E-03	8.333E-04	10.32	10
65		95	650	627	124	62	9.099E-03	9.646E-04	9.43	
66	Ti6Al4V	297	500	1865	260	130	2.752E-02	3.730E-03	7.38	12
67		280	1200	1744	162	81	1.965E-02	1.453E-03	13.52	13
68		280	400	1762	278	139	2.883E-02	4.405E-03	6.54	14,15
69		280	800	1747	198	99	2.270E-02	2.184E-03	10.40	
70		280	1000	1749	176	88	2.086E-02	1.749E-03	11.93	
71		280	1500	1740	146	73	1.821E-02	1.160E-03	15.70	
72		370	1200	2298	184	92	2.154E-02	1.915E-03	11.25	
73		325	1200	2026	176	88	2.086E-02	1.688E-03	12.36	
74		100	1200	629	96	48	1.332E-02	5.242E-04	25.41	
75		165	1200	1031	124	62	1.614E-02	8.592E-04	18.79	
76		370	1000	2295	204	102	2.319E-02	2.295E-03	10.10	
77		120	220	781	240	120	2.602E-02	3.550E-03	7.33	16
78		140	231	907	258	129	2.737E-02	3.926E-03	6.97	
79		140	509	888	176	88	2.086E-02	1.745E-03	11.96	
80		42	200	311	194	97	2.237E-02	1.555E-03	14.39	17
81		42	100	358	254	127	2.707E-02	3.580E-03	7.56	
82		50	200	344	158	79	1.929E-02	1.720E-03	11.22	18
83		75	400	486	142	71	1.784E-02	1.215E-03	14.69	
84		100	600	638	136	68	1.728E-02	1.063E-03	16.26	
85		125	800	786	132	66	1.691E-02	9.825E-04	17.21	
86		150	1000	942	128	64	1.653E-02	9.420E-04	17.54	19
87		175	1200	1093	128	64	1.653E-02	9.108E-04	18.15	
88		195	1200	1218	136	68	1.728E-02	1.015E-03	17.03	
89		195	225	1251	310	155	3.108E-02	5.560E-03	5.59	
90		215	50	1467	664	332	5.160E-02	2.934E-02	1.76	--
91		215	100	1413	482	241	4.190E-02	1.413E-02	1.76	
92		215	150	1393	396	198	3.670E-02	9.290E-03	2.93	
93		42	50	292	146	73	1.821E-02	5.840E-03	3.95	17

Comparison with a multi-physics model:

Figure S1 shows the temperature fields and molten pool geometry on the longitudinal section during LPBF of stainless steel 316 using 300 W laser power and 1250 mm/s scanning speed. Figure S1 (a) shows the result computed using the mechanistic model employed in this work. Essentially, the result corresponds to the top view shown in Figure 3 (a) of the main article. Figure S1 (b) shows the result calculated using a multi-physics, 3D, transient heat transfer and fluid flow model [20]. In both figures, the isotherms are elongated near the trailing edge of the molten pool due to rapid scanning. In Figure S1 (b), the black vectors represent the convective flow of the molten metal. The velocity magnitudes can be estimated by comparing their lengths with the reference vector provided. The convective flow mixes the hot and cold liquids and significantly change the temperature inside the molten pool. However, the molten pool geometries computed using the two models are similar. Since, the susceptibility to gas porosity depends on the pool geometry, the mechanistic model is useful for this purpose for a time-efficient prediction.

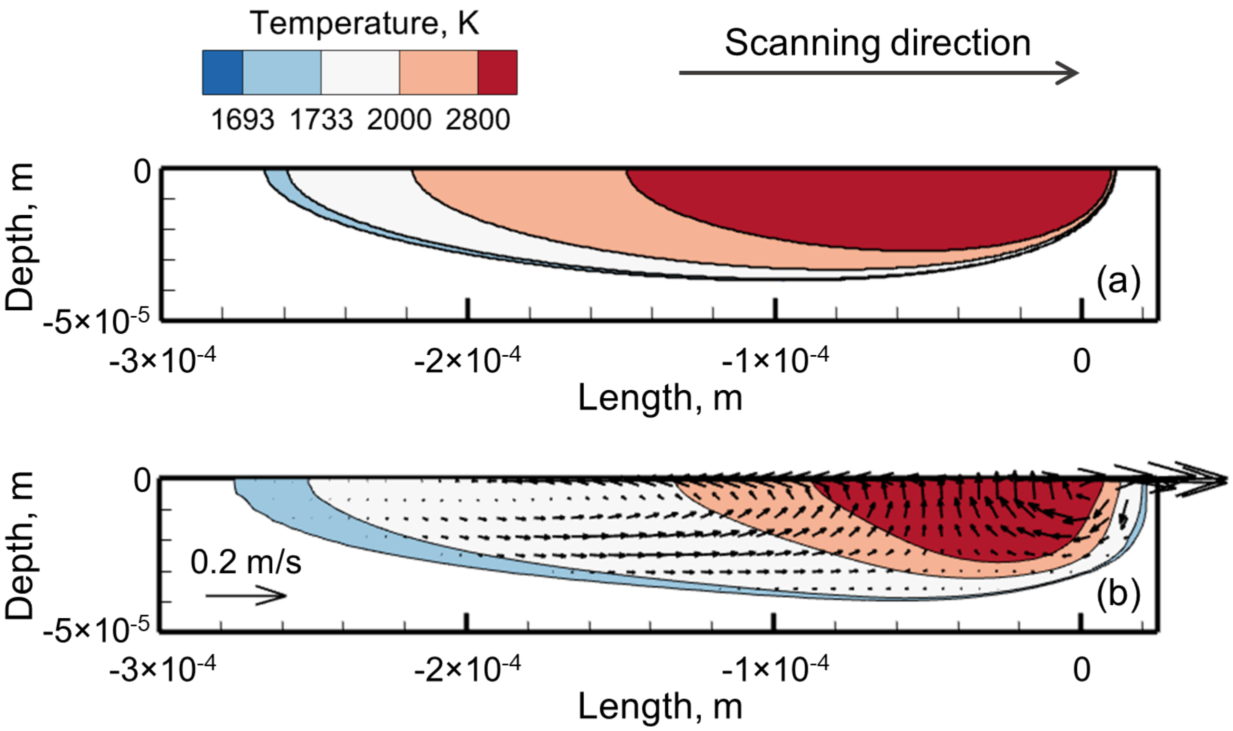

Figure S1: Temperature fields and molten pool geometry on the longitudinal section during LPBF of stainless steel 316 using 300 W laser power and 1250 mm/s scanning speed calculated using (a) the mechanistic model used in this work and (b) a multi-physics, 3D, transient heat transfer and fluid flow model [20].

Supplementary References

1) Mills, K. C. (2002). Recommended values of thermophysical properties for selected commercial alloys. Woodhead Publishing.
2) DebRoy, T., Wei, H. L., Zuback, J. S., Mukherjee, T., Elmer, J. W., Milewski, J. O., ... & Zhang, W. (2018). Additive manufacturing of metallic components–process, structure and properties. Progress in Materials Science, 92, 112-224.
3) Wei, H. L., Mukherjee, T., Zhang, W., Zuback, J. S., Knapp, G. L., De, A., & DebRoy, T. (2021). Mechanistic models for additive manufacturing of metallic components. Progress in Materials Science, 116, 100703.
4) Reijonen, J., Revuelta, A., Riipinen, T., Ruusuvuori, K., & Puukko, P. (2020). On the effect of shielding gas flow on porosity and melt pool geometry in laser powder bed fusion additive manufacturing. Additive Manufacturing, 32, 101030.
5) Diaz Vallejo, N., Lucas, C., Ayers, N., Graydon, K., Hyer, H., & Sohn, Y. (2021). Process optimization and microstructure analysis to understand laser powder bed fusion of 316l stainless steel. Metals, 11(5), 832.
6) Riener, K., Oswald, S., Winkler, M., & Leichtfried, G. J. (2021). Influence of storage conditions and reconditioning of AlSi10Mg powder on the quality of parts produced by laser powder bed fusion (LPBF). Additive Manufacturing, 39, 101896.
7) Kempen, K., Thijs, L., Van Humbeeck, J., & Kruth, J. P. (2015). Processing AlSi10Mg by selective laser melting: parameter optimisation and material characterisation. Materials Science and Technology, 31(8), 917-923.
8) Masiagutova, E., Cabanettes, F., Sova, A., Cici, M., Bidron, G., & Bertrand, P. (2021). Side surface topography generation during laser powder bed fusion of AlSi10Mg. Additive Manufacturing, 47, 102230.
9) Engelhardt, A., Kahl, M., Richter, J., Krooß, P., Kroll, A., & Niendorf, T. (2022). Investigation of processing windows in additive manufacturing of AlSi10Mg for faster production utilizing data-driven modeling. Additive Manufacturing, 55, 102858.
10) Khademzadeh, S., Gennari, C., Zanovello, A., Franceschi, M., Campagnolo, A., & Brunelli, K. (2022). Development of micro laser powder bed fusion for additive manufacturing of Inconel 718. Materials, 15(15), 5231.
11) Tran, H. C., Lo, Y. L., Le, T. N., Lau, A. K. T., & Lin, H. Y. (2022). Multi-scale simulation approach for identifying optimal parameters for fabrication ofhigh-density Inconel 718 parts using selective laser melting. Rapid Prototyping Journal, 28(1), 109-125.
12) Vastola, G., Pei, Q. X., & Zhang, Y. W. (2018). Predictive model for porosity in powder-bed fusion additive manufacturing at high beam energy regime. Additive Manufacturing, 22, 817-822.
13) Narra, S. P., Rollett, A. D., Ngo, A., Scannapieco, D., Shahabi, M., Reddy, T., ... & Lewandowski, J. J. (2023). Process qualification of laser powder bed fusion based on processing-defect structure-fatigue properties in Ti-6Al-4V. Journal of Materials Processing Technology, 311, 117775.
14) Gordon, J. V., Narra, S. P., Cunningham, R. W., Liu, H., Chen, H., Suter, R. M., ... & Rollett, A. D. (2020). Defect structure process maps for laser powder bed fusion additive manufacturing. Additive Manufacturing, 36, 101552.
15) Cunningham, R., Narra, S. P., Montgomery, C., Beuth, J., & Rollett, A. D. (2017). Synchrotron-based X-ray microtomography characterization of the effect of processing

variables on porosity formation in laser power-bed additive manufacturing of Ti-6Al-4V. Jom, 69, 479-484.

16) Wu, Z., Asherloo, M., Jiang, R., Delpazir, M. H., Sivakumar, N., Paliwal, M., ... & Mostafaei, A. (2021). Study of printability and porosity formation in laser powder bed fusion built hydride-dehydride (HDH) Ti-6Al-4V. Additive Manufacturing, 47, 102323.
17) Jaber, H., & Tünde, K. (2020). Development of Selective Laser Melting of Ti6Al4V Alloy for Tissue Engineering.
18) Sanaei, N., & Fatemi, A. (2020). Analysis of the effect of internal defects on fatigue performance of additive manufactured metals. Materials Science and Engineering: A, 785, 139385.
19) Emminghaus, N., Paul, J., Hoff, C., Hermsdorf, J., & Kaierle, S. (2022). Development of an empirical process model for adjusted porosity in laser-based powder bed fusion of Ti-6Al-4V. The International Journal of Advanced Manufacturing Technology, 118(3-4), 1239-1254.
20) Mukherjee, T., Wei, H.L., De, A., & DebRoy, T. (2018). Heat and fluid flow in additive manufacturing—Part I: Modeling of powder bed fusion. Computational Materials Science, 150, 304-313.
